# Association between Age at Menarche and Metabolic Syndrome in Southwest Iran: A Population-Based Case-Control Study

**DOI:** 10.34172/jrhs.2022.93

**Published:** 2022-10-19

**Authors:** Zahra Rahimi, Nader Saki, Bahman Cheraghian, Sara Sarvandian, Seyed Jalal Hashemi, Jamileh Kaabi, Amal Saki Malehi, Arman Shahriari, Nahal Nasehi

**Affiliations:** ^1^Hearing Research Center, Clinical Sciences Research Institute, Department of Biostatistics and Epidemiology, School of Public Health, Ahvaz Jundishapur University of Medical Sciences, Ahvaz, Iran; ^2^Alimentary Tract Research Center, Clinical Sciences Research Institute, Department of Biostatistics and Epidemiology, School of Public Health, Ahvaz Jundishapur University of Medical Sciences, Ahvaz, Iran; ^3^Clinical Sciences Research Institute, Ahvaz Jundishapur University of Medical Sciences, Ahvaz, Iran; ^4^Department of Biostatistics and Epidemiology, School of Public Health, Ahvaz Jundishapur University of Medical Sciences, Ahvaz, Iran; ^5^Alimentary Tract Research Center, Department of Internal Medicine, School of Medicine, Ahvaz Jundishapur University of Medical Sciences, Ahvaz, Iran; ^6^Fertility, Infertility and Perinatology Research Center, Department of Obstetrics and Gynecology, School of Medicine, Ahvaz Jundishapur University of Medical Sciences, Ahvaz, Iran

**Keywords:** Case-control study, Iran menarche, Metabolic syndrome

## Abstract

**Background:** Age at menarche affects women’s health outcomes and could be a risk factor for some diseases, such as metabolic syndrome (MetS). We assessed the association between age at menarche and MetS components in women aged 35-70 in Hoveyzeh, southwest Iran.

**Study Design: **A case-control study

**Methods:** This case-control study was conducted on 5830 women aged 35-70 years in the Hoveyzeh cohort study (HCS), a part of the PERSIAN cohort study, from 2016-2018. The case group included women with MetS, while the controls were women without MetS. The MetS is determined based on standard NCEP-ATP III criteria. Data from demographic, socioeconomic, and reproductive history were gathered face-to-face through trained interviews. Moreover, laboratory, anthropometrics, and blood pressure measurements were assayed for participants. Multiple logistic regression was used to estimate the association between age at menarche and MetS, with adjustment for potential confounding variables.

**Results:** The mean age at menarche was 12.60 ± 1.76 years old. Urban and rural women differed in age at menarche (12.58 ± 1.71 and 12.63 ± 1.83 years, respectively). The study revealed a statistically significant relationship between MetS and menarche age. The odds of developing MetS were 14% higher in women with menstrual age ≤ 11 years than in other groups.

**Conclusion:** As evidenced by the results of this study, the odds of having MetS were higher in women whose menarche age was ≤ 11 years. Furthermore, the association between MetS components and age groups at menarche was statistically significant.

## Background

 Menarche, which is the first menstrual period in a woman’s life, is a marker of female puberty, as well as the onset of ovarian and other endocrine functions related to reproductive capacity.^1,2^ In recent decades, the mean age of menarche has decreased.^1^ Early menarche is often defined as menarche ≤ 11 years old; nonetheless, some investigators base definition on menarche at ≤ 12 years.^3^ Numerous factors, including race, genetics, geographical location, climate, lifestyle, socioeconomic status (SES), physical activity, and nutrition, affect the early or late age of menarche.^4^ Bratke et al proposed a downward trend in the mean age at menarche resulting from an increase in the prevalence of overweight and obesity.^5^

 Age at menarche affects women’s health outcomes^6^; however, the conducted studies have yielded contradictory results. Moreover, different reports have been presented on the average age of menarche in many countries. Some studies demonstrated that age at menarche could be a risk factor for such diseases as metabolic syndrome (MetS),^7^ ovarian cancer,^8^ blood pressure,^9^ type 2 diabetes (T2D),^10^ gestational diabetes mellitus,^11^ and cardiovascular disease (CVD).^12^ Nevertheless, menarche age showed no statistically significant relationship with diabetes^6^ and MetS^2^ in other studies.

 The MetS components include hypertension, hyperglycemia, elevated triglycerides (TG), low high-density lipoprotein cholesterol (HDL-C), and abdominal obesity, a major predictive variable for CVDs.^2^ Over the past two decades, the prevalence of MetS has increased significantly, especially in developing countries.^13^ A systematic review reported the overall prevalence of MetS at 29% in Iran.^14^ The results of the Hoveyzeh cohort study (HCS) demonstrated that the prevalence of MetS was 39.1% (29.3% in men vs. 45.7% in women).^15^ Age, weight, menopause, age at menarche, and genetic factors might be the risk factors for MetS development.^16^ Previous studies have yielded inconsistent results regarding the relationship between age at menarche and MetS. A thorough understanding of this association can be of great help in the development of therapeutic and preventive interventions, as well as the reduction of MetS and other CVDs. In light of the present study, the present study aimed to assess the association of age at the onset of menarche with MetS and its components in women aged 35-70 years in HCS.

## Methods

###  Study design and participants

 This unmatched case-control study was conducted on women aged 35-70 years who entered the enrolment phase of the HCS, which is a population-based cohort study conducted in Hoveyzeh county. This study assesses NCDs in the Arab ethnicity in southwest Iran in 10 009 adults (age 35-70 years) that were recruited from May 2016-August 2018.^15^ The HCS center is one of the sites of the Prospective Epidemiological Research Studies in Iran (the PERSIAN Cohort Study), including 180 000 Iranian adults. All the registration, implementation, and quality control steps of this study have been performed according to the PERSIAN protocol.^17^

 In this study, we selected all participants in the HCS (n = 5983). The participants were asked about their age at the first menstrual. The women who did not have information about menarche age (n = 6) or whose MetS criteria were not measured (n = 147) were excluded from the study. Finally, 5830 women entered into the analysis. The participants were assigned to two groups: MetS as cases (n = 2660) and without MetS as controls (n = 3170). Thereafter, the relationship between the presence or absence of MetS and the age at menarche in the two groups was evaluated. Outcome and exposures were measured equally in the two groups. Informed written consent was obtained from the participants on the day of registration.

###  Metabolic syndrome definition

 Based on the National Cholesterol Education Program-Adult Treatment Panel III guidelines (NCEP ATP3),^18^ participants with three or more of the five below criteria were diagnosed as MetS: Abdominal obesity (waist circumstance ≥ 102 in men and ≥ 88 in women), serum TG ≥ 150 mg/dL, take hypertriglyceridemia medications, HDL < 40 mg/dL in men and < 50 in women, taking drugs for low HDL-C, blood pressure (BP) ≥ 130/85 mm Hg, or taking hypertension drugs, fasting blood sugar (FBS) ≥ 100 mg/dL, and taking hyperglycemia drugs.

###  Assessment of confounders and covariates 

 Three lifestyle measurements were evaluated in the present study. The participant’s physical activity levels by the International Physical Activity Questionnaire (IPAQ)^19^ based on the metabolic equivalent of the task (MET Index). One MET is the energy cost of sitting quietly, equivalent to a caloric consumption of one kcal/kg/h. The person who has smoked at least 100 cigarettes during a lifetime is defined as a smoker.^20^ Another lifestyle measurement was the use of alcohol (yes/no). The wealth index is one of the socioeconomic indexes calculated on interview day according to the information on households’ assets, including freezer, TV, motorbike, cell phone, car, vacuum cleaner, internet access, washing machine, computer, as well as household utilities consisting of house ownership and the number of rooms per capita. These scores were converted into 5-ordered categories, including poorest, poor, moderate, rich, and richest, based on the quintiles.^21^ The assessment of anthropometric measurements was performed while the participants were wearing light clothes and no shoes. Height was measured to the nearest 0.5 cm by a stadiometer (Seca 206), and weight was measured to the nearest 1 kg using a standing scale (Seca 755). Seca locked tape meters measured waist, hip, and wrist circumferences (cm). The body mass index (BMI) was calculated as weight (kg) divided by squared height (m^2^). BP was measured by Riester sphygmomanometers twice (10 minutes intervals) on each arm based on the PERSIAN cohort protocol.^17^Other anthropometric measurements were waist circumference (cm), hip circumference (cm), and wrist circumference (cm).

 Assessment of biological measurements: In the HCS, 25 cc fasting blood was collected from each participant. Blood samples were centrifuged (Sigma, Germany) at 3000 rpm for 10 minutes to separate the serum. Thereafter, the required serum levels were measured by BT 1500 autoanalyzer (Biotecnica Instruments, Italy). The hematology autoanalyzer did a complete blood count (CBC) (Nihon Kohden 6510-k, Japan). Urine tests (urine pH, specific gravity) and analysis were also performed.^17^ Other confounders used contraceptives (yes/no), gravity, parity, and liver enzymes, such as alanine transaminase (ALT), aspartate transaminase (AST), and alkaline phosphatase (ALP).

###  Statistical analysis 

 The mean and standard deviation (SD) for continuous variables, as well as the number and percent for categorical variables, were used to describe the variables. The chi-square test compared the categorical variables. The independent *t *test was used to compare continuous variables between the two groups. Multiple logistic regression was used to assess the association between age at menarche and MetS by controlling confounder factors. The analyses were carried out in SPSS software (version 23). A *P* value was less than 0.05 was considered statistically significant.

## Results

 In this study, we recruited 5830 female participants of the HCS. The mean age at menarche was 12.60 ± 1.76. The mean age at menarche slightly differed in Urban and rural women (12.58 ± 1.71 and 12.63 ± 1.83 years, respectively). Based on the wealth index, the prevalence of early menarche ( ≤ 11 years) in the poorest groups is more than that in the richest groups (22.1% vs. 16.6%). The overall prevalence of MetS in the enrollment phase in women aged 35-70 years in the HCS was 45.62%.^15^ The characteristics of women with or without MetS were reported in Table 1.

**Table 1:** Characteristics of the study population by metabolic syndrome

**Continuous variables**	**Cases (n=2660)**		**Controls (n=3170)**		***P *** **value**
	**Mean**	**SD**	**Mean**	**SD**	
Age (y)	51.12	9.17	46.28	8.54	0.001
Age at menarche (y)	12.51	1.78	12.67	1.74	0.001
Height (cm)	158.97	5.66	158.77	5.68	0.180
Weight (kg)	78.87	13.95	72.00	15.11	0.001
Body mass index (kg/m^2^)	31.18	5.31	28.52	5.60	0.001
Waist circumference (cm)	105.67	10.33	97.73	12.28	0.001
Hip circumference (cm)	107.90	10.07	104.89	10.33	0.001
Wrist circumference (cm)	17.47	1.34	16.93	1.29	0.001
Systolic blood pressure (mm Hg)	116.43	20.06	106.08	15.53	0.001
Diastolic blood pressure (mm Hg)	72.59	11.42	67.82	10.02	0.001
Plus rate	80.47	9.66	78.78	9.27	0.001
Fasting blood sugar (mg/dL)	130.61	60.75	96.37	28.98	0.001
High-density lipoprotein cholesterol (mg/dL)	48.21	10.93	57.17	11.82	0.001
Triglyceride (mg/dL)	197.47	103.05	111.57	46.97	0.001
Total cholesterol (mg/dL)	195.74	43.31	186.93	37.93	0.001
Aspartate transaminase	17.79	11.32	17.22	7.71	0.020
Alanine transaminase	19.21	12.67	16.76	11.54	0.001
Alkaline phosphatase	224.87	65.71	197.64	63.41	0.001
Gravity	6.64	3.15	5.70	2.87	0.001
Parity	6.07	2.70	5.24	2.47	0.001
**Categorical variables**	**Number**	**Percent**	**Number**	**Percent**	***P *** **value**
Wealth index					0.002
Poorest	568	21.35	740	23.34	
Poor	529	19.89	736	23.21	
Moderate	546	20.53	587	18.52	
Rich	537	20.19	586	18.48	
Richest	480	18.04	521	16.43	
Use Alcohol					0.300
Yes	4	0.15	2	0.06	
No	2656	99.85	3168	99.94	
Smoker					0.003
Yes	228	8.57	206	6.50	
No	2432	91.43	2964	93.50	
Education levels					0.001
Illiterate	2132	80.15	2324	73.31	
Primary school	333	12.52	471	14.86	
Secondary school	73	2.74	133	4.20	
Diploma	74	2.79	124	3.91	
University	48	1.80	118	3.72	
Use Contraceptive					0.010
Yes	1582	59.47	1987	62.68	
No	1078	40.53	1183	37.32	

 Participants with MetS were older than those without MetS. Women with MetS had a less-than-mean age at menarche and HDL level. On the contrary, women with MetS had a greater mean BMI, weight, waist circumference, hip circumference, systolic blood pressure (SBP), diastolic blood pressure (DBP), total cholesterol (TC), TG, pulse rate (PR), FBS, ALK, AST, and ALT levels. The chi-square test results pointed to the significant relationship of MetS with wealth index, smoking, education, and contraceptive use (*P* < 0.0001). Furthermore, the results demonstrated that the groups with and without MetS were significantly different in the mean age of menarche, mean weight, waist circumference, hip circumference, waist circumference, SBP, DBP, PR, TC, TG, FBS, ALK, AST, ALT, gravity, and parity (*P* < 0.0001) (Table 1). The MetS and its components were significantly associated with age at menarche (Table 2).

**Table 2:** Metabolic syndrome components in women aged 35-70 years old in the Hoveyzeh Cohort Study

**Age at menarche (y)**	**≤11**		**12-13**		**14-15**		**≥16**		***P *** **value**
	**Number**	**Percent**	**Number**	**Percent**	**Number**	**Percent**	**Number**	**Percent**	
Metabolic syndrome	671	25.2	1205	45.3	700	26.3	84	3.2	0.002
Triglyceride ( ≥ 150 mg/dL) + treatment	500	22.0	1063	46.7	629	27.7	82	3.6	0.025
High-density lipoprotein cholesterol ( < 50 mg/dL) + treatment	637	25.8	1098	44.5	661	26.8	72	2.9	0.001
Blood pressure ( ≥ 130/85 mm Hg) + treatment	429	26.6	703	43.6	439	27.1	43	2.7	0.004
Fasting blood sugar ( ≥ 100 mg/dL) + treatment	639	25.8	1115	45.0	653	26.3	73	2.9	0.007
Waist circumstance ( ≥ 88 cm)	1219	24.0	2307	45.5	1387	27.3	161	3.2	0.001

Table 3 displays the odds ratio (OR) and 95% confidence interval (CI) for MetS according to age at menarche of the women in the four groups ( ≤ 11 years, 12-13 years, 14-15 years, ≥ 16 years), using age at menarche 12-13 years as the reference category. Model 1 depicts crude ORs of MetS for four menarche age groups so that the odds of MetS at menstrual age of ≤ 11 years is 1.14 (95% CI: 1.00, 1.30) more than the reference group. Model 2 is adjusted for demographic variables (age, education), lifestyle (smoking and physical activity), and BMI. The odds of having MetS at menstrual age of ≤ 11 years was 1.04 (95% CI: 0.90, 1.20) more than the reference group. Model 3 is adjusted for biological measurements (FBS, TG, TC, HDL, AST, ALT, and ALK), as well as DPB, SPB, and PR.

**Table 3:** Odds ratios (OR) and 95% confidence intervals (CI) for MetS by menarche age in participants

**Menarche (y)**	**Model 1**			**Model 2**			**Model 3**			**Model 4**			**Model 5**		
	**OR**	**95% CI**	***P*** ** value**	**OR**	**95% CI**	***P*** ** value**	**OR**	**95% CI**	***P*** ** value**	**OR**	**95% CI**	***P*** ** value**	**OR**	**95% CI**	***P*** ** value**
≤ 11	1.14	1.00, 1.30	0.052	1.04	0.90, 1.20	0.588	1.33	1.10, 1.60	0.003	1.17	1.02, 1.35	0.023	1.23	1.01, 1.51	0.038
12-13	Ref.			Ref.			Ref.			Ref.			Ref.		
14-15	0.86	0.76, 0.97	0.017	0.90	0.79, 1.03	0.136	0.96	0.80, 1.14	0.637	0.86	0.76, 0.98	0.026	1.04	0.85, 1.26	0.709
≥ 16	1.01	0.75, 1.36	0.952	1.02	0.74, 1.41	0.923	1.10	0.71, 1.73	0.664	1.04	0.76, 1.44	0.790	1.22	0.74, 2.02	0.429

**Model 1.** Unadjusted. **Model 2:** Adjusted demographic variables (Age, Education), BMI, AND Lifestyle (smoking and Physical activity). **Model 3:** Adjusted biological FBS, measurements (TG, TCHOL, HDL, SGOT, SGPT, ALK), AND DPB, SPB, and PR. **Model 4:** Adjusted Reproductive variable (Gravidity, parity, gestational diabetes and pregnancy hypertension, use of oral contraceptives, and use of hormone replacement therapy). **Model 5:** Adjusted Model 2, Model 3, and Model 4

 The odds of having MetS at menstrual age of ≤ 11 years was 1.33 (95% CI: 1.10, 1.60) more than the reference group. Moreover, after adjustment for reproductive variables (gravidity, parity, gestational diabetes, pregnancy hypertension, use of oral contraceptives, and use of hormone replacement therapy), model 4 demonstrated the odds of having MetS in groups with menstrual age ≤ 11 years (95% CI: 1.02, 1.35) was 14% higher than that in the reference group (Table 3). Adjusted Model 2, Model 3, and Model 4 reported in model 5, the odds of having MetS at menstrual age of ≤ 11 years was 1.23 (95% CI: 1.01, 1.51) more than the reference group.

## Discussion

 Based on previously conducted studies, the age of menarche affects health outcomes in the later years of women’s lives. In the current study, the age of menarche was not much different from the average age of girls in Iran. The age at menarche in this study was lower than that in a previous study in Khuzestan province, and this downward trend can be due to the increase in obesity and hormonal changes in children.^22^ The mean age at menarche has been reported differently in studies conducted across the globe. The result of the present study demonstrated that the mean age at menarche in Iran is similar to that in some countries, such as the United States and Turkey, and less than that in Indian and Chinese women.^12,23-25^

 The warm climate of Hoveyzeh in southwestern Iran might be the reason for the lower mean age of menarche in this area than that in women who live in cold regions.^26^ This study demonstrated that the mean age at menarche in urban regions is slightly lower than that in rural areas. Different studies almost confirmed this result and pointed to the association of the urban/rural setting with early menarche. This difference in menarche age can be attributed to overnutrition, obesity, and overweight in urban girls.^27,28^

 In the current study, rich women experienced menarche earlier. In line with our results, other studies in Mexico reported that women with high SES reached menarche the earliest and those with low SES the latest. The historical decline in age at menarche for high and medium SES groups occurred relatively early, whereas it was observed later in low SES groups. Moreover, the US study found income status related to the timing of menarche over time when the proportion of girls experiencing early menarche ( < 11 years old) increased over 50 years only for those girls of a low socioeconomic position.^29^ On the contrary, another study in the United Kingdom spanning 85 years found a decline in the timing of menarche across all socioeconomic groups.^30^

 The results of the current study pointed to a relationship between MetS and the age of menarche at the age of below 11 years; nonetheless, different studies have yielded inconsistent results. This discrepancy might be due to differences in the study area and participants, assimilation in confounding variables, study design, and sample size.^22^ The results of studies on the relationship between menarche age and MetS are not definite.^31,32^ In agreement with the present research, some studies demonstrated an association between age at menarche in women with and without MetS.^33,34^ The most suggested explanation is that early menarche in adolescents and MetS in adulthood are the consequences of childhood obesity.^32^ A study conducted on Chinese women reported that the age at menarche was inversely associated with the total number of MetS components.^35^ Cho et al showed no association between age at menarche and MetS.^31^

 Lee et al suggested that the risk of MetS was significantly increased at ≤ 12 years of age at menarche (OR = 1.91, *P* < 0 05).^1^ Glueck et al indicated that menarche age had a curvilinear (‘U’ shaped) relationship with MetS in adulthood. Late and early menarche are risk factors for MetS and cardiometabolic abnormalities in adults.^36^ Moreover, Hwang et al reported that early or late menarche was not associated with an increased risk of MetS in premenopausal Korean women.^2^

 The present study illustrated a statistically significant relationship between the component of MetS and menarche age groups. Janghorbani et al showed a strong association between younger age at menarche and an increased risk of T2D.^37^ A study in Brazil pointed to a relationship between menarche age (less than 11 years) and a high risk of diabete.^38^ Sumi et al, in a retrospective cohort study on the relationship of menarche age with obesity and glycemic control in T2D demonstrated that age at menarche was inversely correlated with BMI and abdominal circumference.^39^ Yang et al reported that menarche age has a linear and inverse relationship with the incidence of diabetes for each year of delay.^40^ Another study by Qiu et al found no significant association between menarche age and diabetes. Nonetheless, there was a significant relationship between late menarche age (18 years) and lower CVD risk.^6^ Lee et al reported that women with age at menarche ≤ 12 years had significantly higher levels of triglycerides. Furthermore, hypertriglyceridemia was significantly increased at early menarche.^1^

 A major strength of the present study is the large study population. Another advantage was the use of high-reliability cohort study data. A limitation of our study is the absence of actual menarche dates of the girls and the use of self-reporting that may be prone to recall bias. However, previous studies pointed out that self-reporting for menarche dates is reliable. Furthermore, we conducted this study on Arab ethnicity, and the results of our study may have low external validity.

**Highlights**

- The mean age at menarche in this study is 12.60 years old.
- The odds of metabolic syndrome are higher in adult women whose menarche age was ≤ 11 years.
- The age at menarche is higher in women living in rural areas and those from the poorest families (based on the wealth index).

## Conclusion

 As evidenced by the obtained results, the odds of having MetS were higher in women whose menarche age was ≤ 11 years. Moreover, the association between MetS components and age groups at menarche was statistically significant. Although determining the average age of menarche can effectively identify people with the risk of MetS, cohort studies are needed to clarify this relationship

## Acknowledgments

 The ethics committee approved this project of Ahvaz Jundishapur University of Medical Sciences (Ethical code: IR.AJUMS.REC.1398.272). This study was performed in accordance with the Helsinki Declaration. Informed consent was obtained from all participants.

## Conflict of interest

 None to declare.

## Funding

 The Iranian Ministry of Health and Medical Education has contributed to the funding used in the Persian Cohort through Grant number 700/534 and the Vice-Chancellor for Research at Ahvaz Jundishapur University of Medical Sciences grant number HCS-9809.
